# Revealing the Role of the Calcineurin B-Like Protein-Interacting Protein Kinase 9 (CIPK9) in Rice Adaptive Responses to Salinity, Osmotic Stress, and K^+^ Deficiency

**DOI:** 10.3390/plants10081513

**Published:** 2021-07-23

**Authors:** Sergey Shabala, Mohammad Alnayef, Jayakumar Bose, Zhong-Hua Chen, Gayatri Venkataraman, Meixue Zhou, Lana Shabala, Min Yu

**Affiliations:** 1 International Research Centre for Environmental Membrane Biology, Foshan University, Foshan 528000, China; 2 Tasmanian Institute of Agriculture, University of Tasmania, Hobart, TAS 7005, Australia; m-nayef@hotmail.com (M.A.); meixue.zhou@utas.edu.au (M.Z.); L.Shabala@utas.edu.au (L.S.); 3 Australian Research Council Centre of Excellence in Plant Energy Biology, School of Agriculture, Food and Wine, University of Adelaide, Glen Osmond, SA 5064, Australia; jayakumar.bose@adelaide.edu.au; 4 Hawkesbury Institute for the Environment, Western Sydney University, Penrith, NSW 2751, Australia; Z.Chen@westernsydney.edu.au; 5 Plant Molecular Biology Laboratory, M.S. Swaminathan Research Foundation, Chennai 600113, India; gayatri@mssrf.res.in

**Keywords:** calcium signalling, potassium transport, AKT, HAK, reactive oxygen species, ABA, stomata, CBL, CIPK

## Abstract

In plants, calcineurin B-like (CBL) proteins and their interacting protein kinases (CIPK) form functional complexes that transduce downstream signals to membrane effectors assisting in their adaptation to adverse environmental conditions. This study addresses the issue of the physiological role of CIPK9 in adaptive responses to salinity, osmotic stress, and K^+^ deficiency in rice plants. Whole-plant physiological studies revealed that *Oscipk9* rice mutant lacks a functional CIPK9 gene and displayed a mildly stronger phenotype, both under saline and osmotic stress conditions. The reported difference was attributed to the ability of *Oscipk9* to maintain significantly higher stomatal conductance (thus, a greater carbon gain). *Oscipk9* plants contained much less K^+^ in their tissues, implying the role of CIPK9 in K^+^ acquisition and homeostasis in rice. *Oscipk9* roots also showed hypersensitivity to ROS under conditions of low K^+^ availability suggesting an important role of H_2_O_2_ signalling as a component of plant adaptive responses to a low-K environment. The likely mechanistic basis of above physiological responses is discussed.

## 1. Introduction

An understanding of plant responses to abiotic stress is vital for the genetic engineering of climate-resilient crops. This involves understanding the mechanisms by which plants sense stresses and generate appropriate stress-induced signals, such as changes in the cytosolic free Ca^2+^ and ROS [1,2,3]. These changes then produce what is known as the “Ca^2+^ signature” [4,5] triggering a protein phosphorylation cascade that finally targets proteins directly involved in cellular protection, or transcriptional factors regulating stress-induced genes [6]. Salinity [7,8,9], drought [10,11], and K^+^ deficiency [12] have all been shown to induce transient Ca^2+^ influx, thereby increasing cytosolic Ca^2+^ concentration. This change in the cytosolic Ca^2+^ levels can be detected by numerous high-affinity calcium sensors. In higher plants, several families of Ca^2+^ sensors have been recognised, including calmodulin (CaM) and CaM-related proteins [13,14], Ca^2+^ dependent protein kinases (CDPKs) [15,16], and calcineurin B-like (CBL) proteins and their interacting protein kinases (CIPKs) [17]. CBL–CIPKs interactions transduce downstream signals to membrane effectors (various membrane ion channels, pumps, and transporters) which, in turn, facilitate ionic homeostasis by controlling ion uptake, long-distance transport, and sequestration [18]. Ten CBL and 26 CIPK isoforms are present in *Arabidopsis*, and in rice these numbers are 10 and 30, respectively [19,20]. *OsCIPKs* genes are highly responsive to external stimuli, with 15, 12, 12, and 16 *OsCIPKs* being induced by drought, salinity, PEG, and ABA treatments, respectively, in rice [21].

The salt overly sensitive (SOS) pathway is one of the classical examples of a CBL–CIPK signalling pathway in response to salt stress. SOS3/CBL4 has been identified as a Ca^2+^ binding protein [17]. In response to salt stress, the transient alleviation in the cytosolic Ca^2+^ concentration activates SOS3/CBL4, which then interacts with SOS2/CIPK24 to directly regulate the downstream component of SOS1, a putative Na^+^/H^+^ antiporter, with the final result being the maintenance of low intracellular Na^+^ [22]. The SOS3–SOS2 complex may also regulate the tonoplast Na^+^/H^+^ antiporter, assisting in compartmentation of toxic Na^+^ in the vacuole, thus lowering Na^+^ concentration in the cytosol [23]. In addition, SOS2/CIPK24 may modulate the plasma membrane H^+^/Ca^2+^ antiporter (CAX1) to control intracellular Ca^2+^ homeostasis [24]. Rice OsCIPK24 and OsCBL4 were also able to activate OsSOS1 in yeast cells [25], although the beneficial effects of such activation on salinity tolerance was questioned [26].

The ability of plants to maintain cytosolic K^+^ homeostasis is critical to confer both salinity and drought tolerance [27,28,29]. The CBL1/CBL9-CIPK23 complex regulates the AKT1 pathways that play a key role in K^+^ homeostasis under water stress. A loss of function of *Atcipk23* and *cbl1/cbl9* results in an increased drought tolerance in *Arabidopsis* resulting from the mutants possessing a hypersensitivity of stomata to ABA, which in turn caused a reduction in transpiration rate [30]. In rice, OsCBL1-OsCIPK23 operates upstream of OsAKT1, and it has been reported that loss of function of *Oscipk23* caused similar symptoms of K^+^ deficiency as occurred in the *Osakt1* mutant under low K^+^ conditions. This suggested the critical role of OsCIPK23 in modulating AKT1 activity in K^+^ homeostasis in rice plants [31]. High affinity K^+^ uptake systems are also regulated by CBL–CIPK interaction [32].

With the large number of members of CBL–CIPK families, not all of them have been properly characterized at the functional level. One of these is CIPK9 (Locus At1G01140). In *Arabidopsis*, *AtCIPK9* expression was ubiquitous in the mature root zone, but less pronounced in the elongation zone [33]. Moreover, its expression was inducible under abiotic stress (osmotic stress; salinity; cold) as well as under low K^+^ conditions [33]. *Atcipk9* mutant plants were hypersensitive to K^+^-deficient conditions [34], most likely as a consequence of impaired AtHAK5-mediated K^+^ uptake [35]. It was also shown that *At*CIPK9 interacts with tonoplast CBL2 and CBL3, to confer K^+^ homeostasis in *Arabidopsis* [36]. Consistent with this, no significant difference in K^+^ uptake or content was observed in plants cultivated both in high K^+^ (20 mM), and low K^+^ (0.02 mM) growth media [33]. Other reported roles of CIPK9 in *Arabidopsis* include responses to wounding [37], regulation of Mg homeostasis [38], and growth under high external Mg^2+^ conditions [39], as well as NH_4_-dependent root growth [40]. Although AtCIPK9 and OsCIPK9 are orthologous by phylogenetic analysis, and have about 79% of identity in protein sequences [41], the role of CIPK9 in regulation of K^+^ homeostasis and responses to abiotic stresses in rice has never been revealed. In this study, we aimed to fill this gap in the knowledge. By conducting a range of whole-plant physiological and cell-based electrophysiological experiments, here we demonstrate that *Oscipk9* rice mutant, lacking a functional CIPK9 gene, displayed a mildly stronger phenotype, both under saline and osmotic stress conditions. This difference was attributed to the ability of *Oscipk9* plants to maintain significantly higher stomatal conductance. OsCIPK9 also played an important role in K^+^ acquisition and homeostasis, with *Oscipk9* roots showing hypersensitivity to ROS under conditions of low K^+^ availability.

## 2. Results

### 2.1. Oscipk9 Mutant Is Mildly Salt-Tolerant

**Figure 1:**
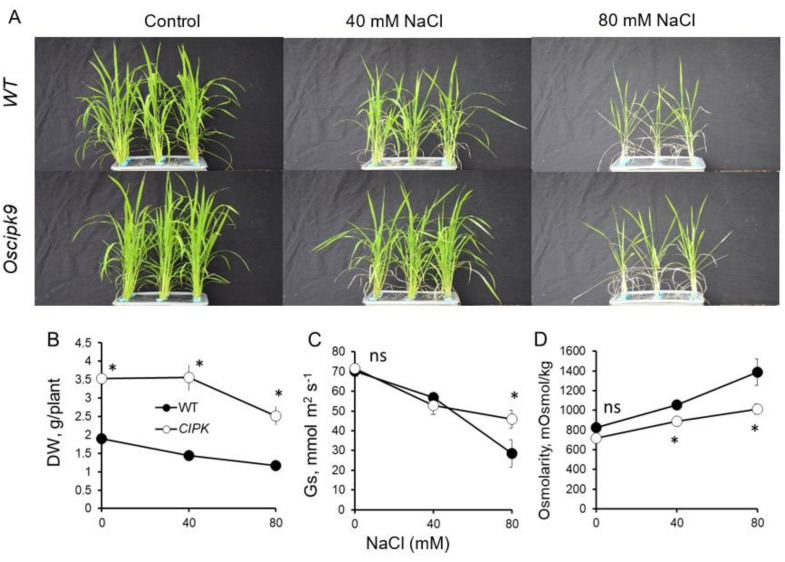
Effects of salinity stress on growth and phenotype of Oscipk9 and WT plants. (**A**) plant phenotype under control, mild (40 mM NaCl for 3 weeks) and severe (80 mM NaCl) treatments; (**B**) plant dry weight; (**C**) stomatal conductance; and (**D**) shoot osmolality. Closed circles—WT; open circles—Oscipk9 mutant line. Data are the mean ± SE (*n* = 18). Asterisk denotes significant difference between WT and cipk9 plants at *p* < 0.05.

The Oscipk9 mutant showed a significantly (by 47%; *p* ≤ 0.01) higher dry weight than the wild type (WT) under control (non-saline condition) (Figure 1A,B). Its dry weight (DW) was not affected by exposure to moderate salinity (40 mM) for 3 weeks, while WT plants showed a significant (~24%; *p* ≤ 0.05) decline (Figure 1B). More severe (80 mM) salinity treatment caused a further reduction in plant DW that was more pronounced in the Oscipk9 mutant. Stomatal conductance was identical for 0 and 40 mM NaCl treatments between two lines, but significantly higher in cipk9 knockout under the high saline condition (Figure 1C) compared with WT. Both lines had the same shoot osmolality under control conditions (Figure 1D) that progressively increased upon salinity exposure. At all concentrations, the osmolality was significantly (*p* ≤ 0.01) lower in the Oscipk9 mutant compared to the WT (Figure 1D). Overall, this data indicates a mildly salt-tolerant phenotype in Oscipk9 mutant.

**Figure 2:**
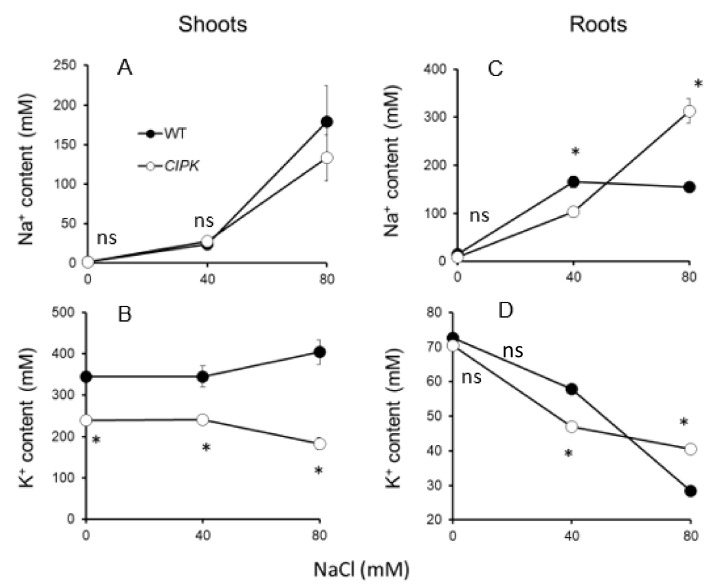
Effect of salinity on plant ionic composition. (**A**) shoot Na^+^ content, (**B**) shoot K^+^ content; (**C**) root Na^+^ content; and (**D**) root K^+^ content. Plants of both lines were grown in a hydroponic system for 21 days under three NaCl levels (0, 40, and 80 mM NaCl). Closed circles—WT; open circles—*Oscipk9* mutant line. Data are the mean ± SE (*n* = 6). Asterisk denotes significant difference between WT and cipk9 plants at *p* < 0.05.

Oscipk9 mutant had a similar shoot Na^+^ (Figure 2A) but much lower (by 43%; significant at *p* ≤ 0.01; Figure 2B) K^+^ content in the shoot when grown under the non-saline control condition, compared with WT. Exposure to salinity for 3 weeks altered both the Na^+^ and K^+^ content in both shoots and roots, in both lines. Under moderate salinity, the shoot Na^+^ content gradually increased, but was not significantly different between the lines (Figure 2A), while the shoot K^+^ content was not affected and remained similar to the control level (Figure 2B). Severe (80 mM NaCl) salinity treatment reduced K^+^ content in Oscipk9, but not in WT shoots (Figure 2B). Shoot Na^+^ content was slightly higher in WT but not significantly (*p*< 0.05) different under severe salinity treatment. No significant differences were detected in root Na^+^ and K^+^ contents between the lines under the non-saline condition (Figure 2C,D). The dose-dependent increase in root Na^+^ content was reported for Oscipk9 plants, while in WT root Na^+^ has “stabilized” at around 150 mM level and did not increase with increasing salinity (Figure 2C). Root K^+^ content declined in both lines in a dose-dependent manner, with no clear difference between Oscipk9 and WT plants (Figure 2D).

### 2.2. Oscipk9 Mutant Performs Better under Osmotic Stress Conditions

**Figure 3:**
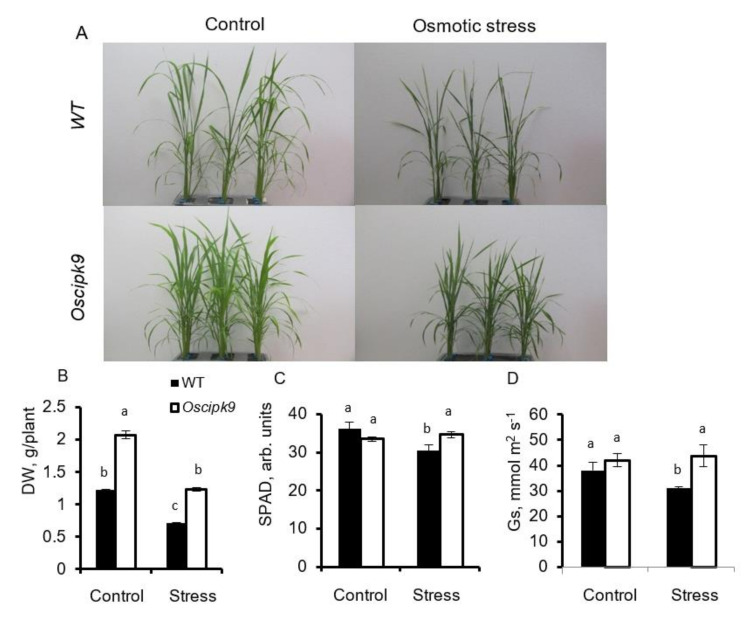
Effect of osmotic stress on growth and phenotype of *Oscipk9* and WT plants. Osmotic stress was induced by adding 11.8% (*w*/*v*) of PEG4000 (imposing an osmotic stress of 0.362 MPa) to the hydroponics growth solution. (**A**) plant phenotype; (**B**) plant dry weight; (**C**) chlorophyl content (SPAD values); and (**D**) stomatal conductance. Closed bars-WT; open bars-*Oscipk9* mutant line. Data are the mean ± SE (*n* = 18). Data labelled with different low-case letters is significantly different at *p* < 0.05.

*Oscipk9* mutant experienced a stronger vegetative growth and produced a larger number of tillers (Figure 3A) resulting in a significantly (~40%; *p* ≤ 0.01) higher dry weight (compared with WT) in plants grown under control conditions (Figure 3B).

**Figure 4:**
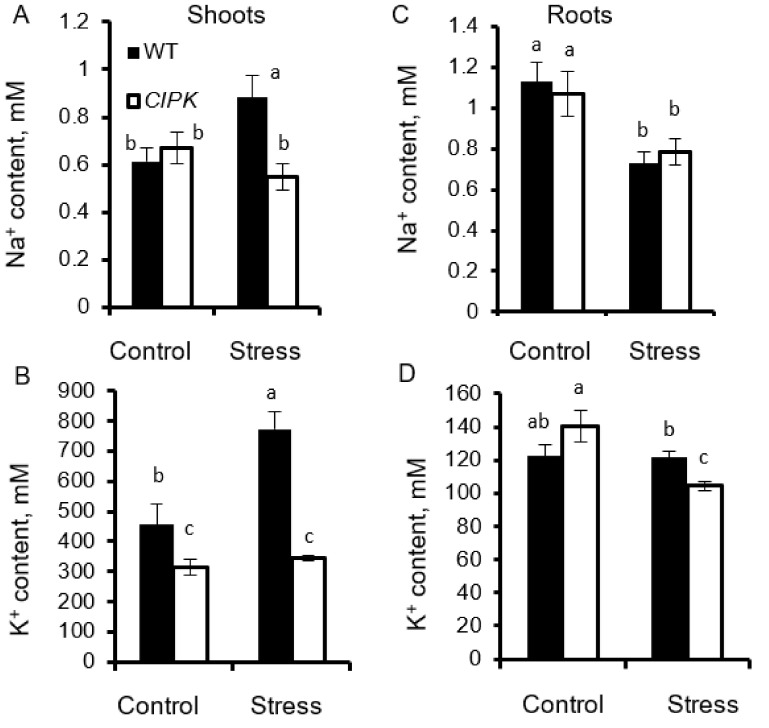
Effect of osmotic stress on plant ionic composition. (**A**) shoot Na^+^ content, (**B**) shoot K^+^ content; (**C**) root Na^+^ content; and (**D**) root K^+^ content. Plants of both lines were grown in a hydroponic system for 21 days and treated with PEG4000. Closed bars—WT; open bars—Oscipk9 mutant line. Data are the mean ± SE (*n* = 6). Data labelled with different low-case letters is significantly different at *p* < 0.05.

No significant (at *p* < 0.0) difference was reported for either leaf chlorophyll content (SPAD values; Figure 3C) or stomatal conductance (Figure 3D) between two lines under control conditions. Osmotic stress (PEG treatment) reduced plant DW in both lines by ~40%, with no significant (*p* < 0.05) difference in relative DW changes between the lines (Figure 3B). Chlorophyll content and stomatal conductance decreased slightly in WT but not Oscipk9 plants (Figure 3C,D, respectively). Shoot Na^+^ and K^+^ contents have both increased under drought conditions in WT, but remained unchanged in Oscipk9 mutant (Figure 4A,B). In roots, osmotic stress reduced Na^+^ content slightly in both lines (Figure 4C), while K^+^ content remained unchanged in WT but declined in stress-exposed Oscipk9 plants (Figure 4D).

### 2.3. Oscipk9 Mutant Is More Sensitive to Low K^+^ Availability

**Figure 5:**
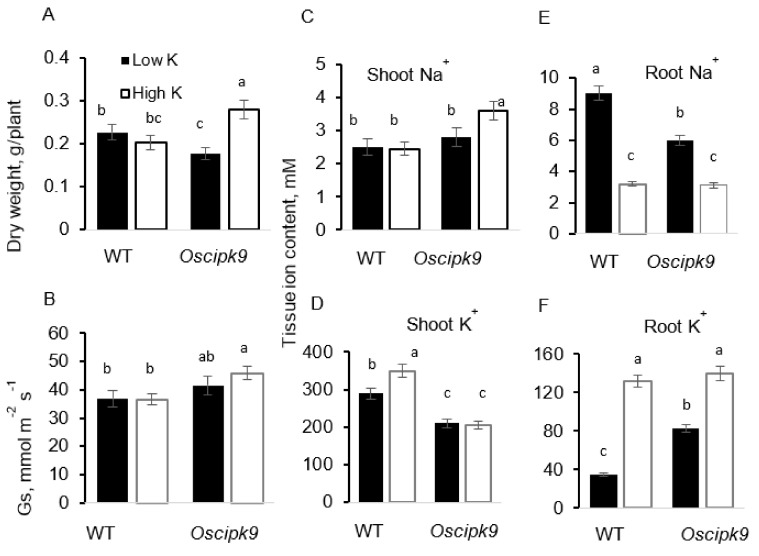
Effects of two K^+^ availability on agronomical and physiological characteristics of WT *Oscipk9* mutant plants. (**A**) plant dry weight; (**B**) stomatal conductance; (**C**) shoot Na^+^ content; (**D**) shoot K^+^ content; (**E**) root Na^+^ content; and (**F**) root K^+^ content. Closed bars—WT; open bars—*Oscipk9* mutant line. Data are the mean ± SE (*n* = 18). Data labelled with different low-case letters is significantly different at *p* < 0.05.

Low K^+^ availability came with the penalty to Oscipk9 mutant growth as compared to WT while these plants were more responsive to high K^+^ (Figure 5A). No significant effects of K^+^ availability on stomatal conductance were found (Figure 5B). Shoot Na^+^ content remained unchanged regarding K^+^ availability in WT, but increased under conditions of K deficiency in Oscipk9 mutant (Figure 5C). Root Na^+^ content decreased dramatically in both lines under luxury K^+^ supply (Figure 5E). Shoot K^+^ content has increased by increasing K^+^ availability in WT but remained unchanged in Oscipk9 mutant (Figure 5D), and K^+^ content was consistently higher in WT. In roots, plants grown under low-K^+^ conditions had significantly (*p* < 0.05) less K^+^ (Figure 5F), with the lowest K^+^ content reported in WT plants.

### 2.4. Roots of Oscipk9 Mutant Are More Sensitive to H_2_O_2_

Both salinity and osmotic stress results in overaccumulation of reactive oxygen species that may affect plant ionic homeostasis (hence, growth). These ROS are also known to interact with Ca^2+^ transport and signalling systems, forming so-called “ROS-Ca^2+^ Hub” [42] by forming a feedback loop between Ca^2+^-permeable plasma membrane channels and NADPH oxidase that generates apoplastic ROS. Given the important role of CIPKs in Ca^2+^ signalling, we have compared the differences in kinetics of ROS-induced net Ca^2+^ and K^+^ fluxes between Oscipk9 and WT plants under conditions of various K^+^ and Ca^2+^ availability.

**Figure 6:**
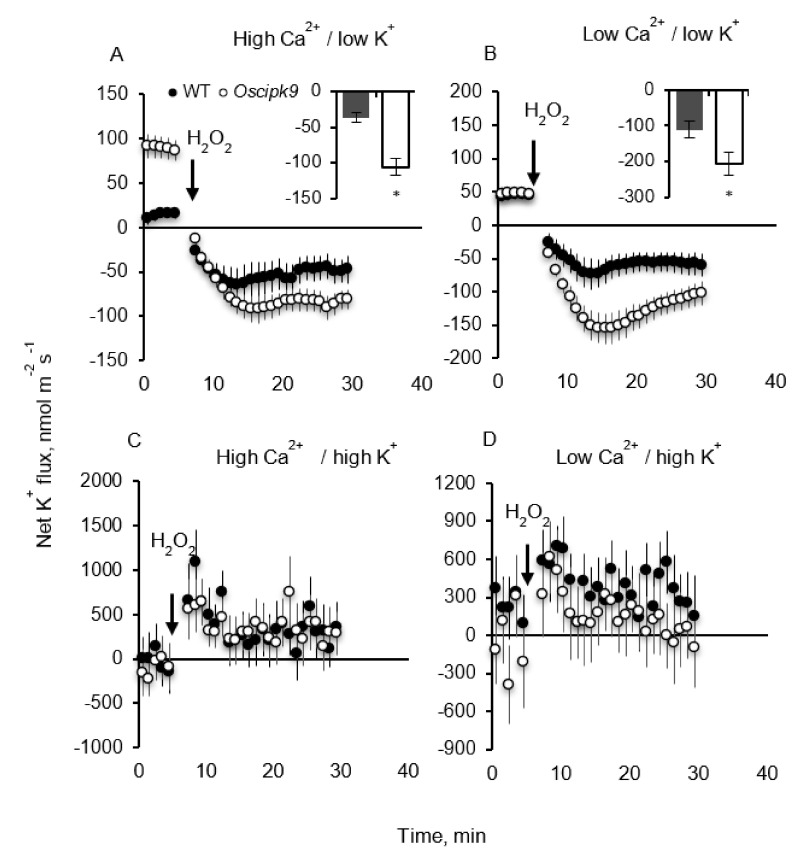
Effect of K^+^ and Ca^2+^ availability on kinetics of ROS-induced net K^+^ efflux measured from epidermal mature root cells of *Oscipk9* mutant and its WT. ROS stress was imposed by adding 5 mM H_2_O_2_ to 5–6-day-old seedlings. (**A**) high Ca/low K (1.5 mM/0.1 mM) conditions; (**B**) low Ca/low K (0.1 mM/0.1 mM) conditions; (**C**) high Ca/high K (1.5 mM/10 mM) conditions; and (**D**) low Ca/high K (0.1 mM/10 mM) conditions. Data are the mean ± SE (*n* = 6). The sign convention is “efflux negative”. The inserts in the top two panels depict the magnitude of responses in each line. The sign convention is “efflux negative”. The inserts in the top two panels depict the magnitude of responses in each line. Open symbols-*Oscipk9*; closed symbols-WT. Asterisk denotes significant difference between WT and cipk9 plants at *p* < 0.05.

Oxidative stress (5 mM H_2_O_2_ treatment) triggered massive K^+^ loss from plant roots under low-K^+^ conditions (Figure 6A,B); this loss was much more pronounced in Oscipk9 mutant (about 2-fold stronger net K^+^ efflux; significant at *p* < 0.05). In WT, these responses were independent of Ca^2+^ availability, while in the mutant, low Ca^2+^ availability increased sensitivity of ROS-activated K^+^-permeable channels to H_2_O_2_. Under luxury K^+^ supply (10 mM) H_2_O_2_ treatment did not cause any net K^+^ loss (Figure 6C,D) but instead triggered a shift towards increased K^+^ uptake. No significant (at *p* <0.05) effects of Ca^2+^ availability were detected in this case.

**Figure 7:**
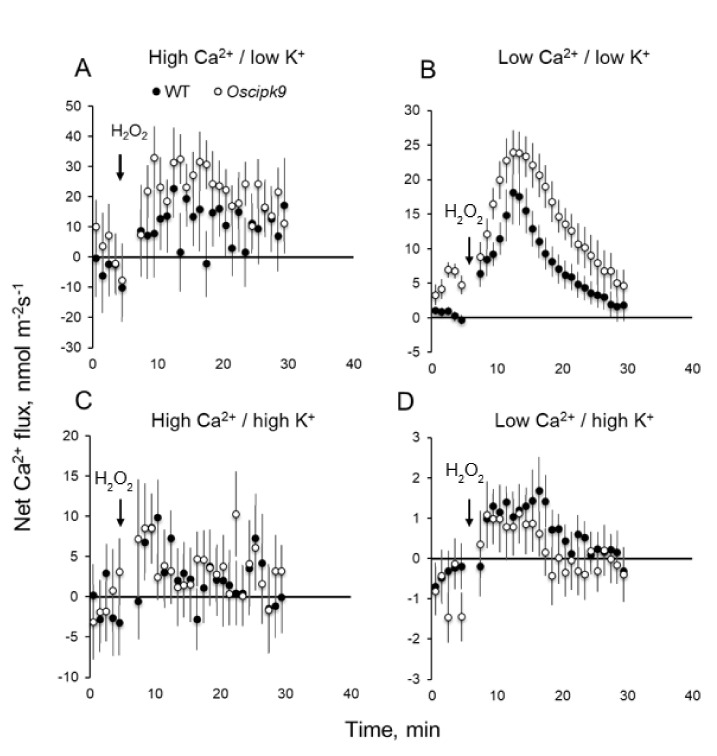
Effect of K^+^ and Ca^2+^ availability on kinetics of ROS-induced net Ca^2+^ fluxes measured from epidermal mature root cells of *Oscipk9* mutant and its WT. ROS stress was imposed by adding 5 mM H_2_O_2_ to 5–6-day-old seedlings. (**A**) high Ca/low K (1.5 mM/0.1 mM) conditions; (**B**) low Ca/low K (0.1 mM/0.1 mM) conditions; (**C**) high Ca/high K (1.5 mM/10 mM) conditions; and (**D**) low Ca/high K (0.1 mM/10 mM) conditions. Open symbols—*Oscipk9*; closed symbols—WT.

ROS treatment has resulted in an increased Ca^2+^ uptake in plant roots (Figure 7). No significant difference was found between ROS-induced Ca^2+^ flux responses under conditions of high-K^+^ supply (Figure 7C,D), while under low-K^+^ conditions, Oscipk9 mutant was more sensitive to ROS treatment (Figure 7A,B).

## 3. Discussion

The CBL–CIPK interacting complexes transduce various developmental and adaptive signals to downstream effectors, thus mediating plants responses to environment. In this work, we have shown that the loss of function in CIPK9 gene resulted in a mildly salt- and osmotic-stress-tolerant phenotype in rice. *Oscipk9* plants accumulated less K^+^ in the shoot (Figure 4) but possessed higher stomatal conductance (Gs) (Figure 2 and Figure 3). *Oscipk9* roots also showed hypersensitivity to ROS under conditions of low K^+^ availability (Figure 6 and Figure 7).

### 3.1. CIPK9 Is Essential for Stomata Operation under Stress Conditions

The loss of functionality in CIPK9 has resulted in mildly salt- (Figure 1) and osmotic (Figure 3) stress-tolerant phenotypes in rice, which could be related to the ability of *Oscipk9* plants to maintain higher Gs values (Figure 1C; Figure 3D) compared with WT; hence, they assimilate more carbon under conditions of reduced water availability.

Stomata represent the microscopic sphincters on the leaf surface that balance CO_2_ intake and water loss [43], and the ability of a plant to optimise stomatal aperture is critical for adaptation to adverse environmental conditions, especially salinity and drought [44]. Each stoma consists of a pair of guard cells, and its aperture is regulated by changes in the guard cell turgor mediated by rapid fluxes of ions into or out of cell [45]. This is achieved by sensing and transducing numerous environmental and internal signals. In the latter case, ABA is arguably the most prominent second messenger controlling stomata aperture [46,47]. It was shown that halophytic and glycophytic species (contrasting in their salinity stress tolerance), possess different baseline ABA levels, and that halophyte stomata are more sensitive to the fluctuation in ABA content in leaf mesophyll [45].

Early studies have suggested that *cipk9-1*, a null mutant of CIPK9, was hypersensitive to ABA during seed germination [48,49]. More recently, Lu et al. [50] have shown that overexpression of NtCIPK9 from *Nitraria tangutorum* in *Arabidopsis* resulted in a higher germination rate in the presence of NaCl, and they attributed this effect to the regulation of endogenous ABA levels in plants. These findings suggest a causal link between CIPK9 operation and ABA production/signalling that could potentially explain higher Gs values in stress-exposed *Oscipk9* plants in our study. It has been shown [51] that the functional loss of the CBL2/3-CIPK9/17 complex in *Arabidopsis* guard cells resulted in ABA hypersensitive stomatal closure and enhanced drought tolerance. This hypersensitive response was attributed to rapid modulation of potassium homeostasis at the tonoplast, presumably via activity of NHX K^+^(Na^+^)/H^+^ exchangers [51] although no supportive evidence was presented. More recently, Tang and co-authors demonstrated that CIPK9 preferentially phosphorylates two of its CBL partners, CBL2 and CBL3, and regulates TPK (two-pore potassium) vacuolar channels involved in remobilization of K^+^ from the vacuole [52]. Taken together, the data suggests that the higher Gs values and mildly tolerant phenotype of *Oscipk9* plants under water-limiting conditions may be a result of the negative regulation of tonoplast K^+^ channels in guard cell vacuoles by CIPK9-CBL2/CBL3 complex, downstream of stress-induced ABA signalling.

### 3.2. CIPK9 Is Essential for Rice Responses to Low K^+^ Availability

Plant K^+^ acquisition is mediated by several low- and high-affinity uptake systems; of these, AKT1 inward-rectifying K^+^ channels and high affinity HAK5 K^+^ transporters are considered to be critical [29]. Both are located at the plasma membrane and activated by CBL–CIPK complexes [53], specifically by CIPK23-CBL1/CBL9 [6,54]. Upon interaction with one of these CBLs, CIPK23 is recruited to the plasma membrane phosphorylates AKT1 K^+^ channel, so that AKT1-mediated root K^+^ uptake is enhanced [54].

CIPK9 is also known as a regulator of K^+^ deficiency [55], and the growth of *Atcipk9* mutants was negatively affected at low (0.01 mM) K^+^ availability suggesting that CIPK9 may function in plant adaptation to K^+^ starvation [33]. *Arabidopsis* plants lacking CIPK9 displayed a tolerant phenotype to low-K stress, and it was shown that CIPK9 interacts with the calcium sensors CBL3 and CBL2 to regulate plant adaptive responses to K^+^ starvation [38,56]. Here, we show that the loss of CIPK9 in rice also compromises plant growth under conditions of low- but not high-K availability (Figure 5A). Thus, although CIPK orthologs from different species can have various roles [50], the essential role of CIPK9 in plant adaptation to K^+^ starvation is preserved amongst multiple species.

### 3.3. CIPK9 Control over K^+^ Translocation and Compartmentalization

Recently, Tang et al. [52] showed that, although *cbl2 cbl3* double mutant plants were extremely sensitive to low-K levels in the medium, they exhibited a significantly higher K content as compared with the wild type, particularly under low-K conditions. These findings suggest that it is K^+^ homeostasis, but not uptake per se, that may be affected by CBL–CIPK interacting complex, suggesting a likely role of CIPK9 in K^+^ translocation between shoots and roots, especially under low-K conditions [36]. Consistent with these suggestions are our findings that while shoot K^+^ was not different between *Oscipk9* plants grown under low- and high-K conditions (Figure 5D), a nearly two-fold difference was observed in plant roots (Figure 5F). It was suggested earlier that the overexpression of CIPK9, CBL2, and CBL3 may impair root K^+^ uptake from the environment [36]; thus, our observation of higher root K^+^ content in the mutant plants lacking functional CIPK9 gene (Figure 5F) are consistent with those reports.

In a stark contrast to CIPK23, that is localized predominantly at the plasma membrane, CIPK9 is associated with a tonoplast [6] and, hence, controls plant K^+^ homeostasis by regulating its transport between vacuolar and cytosolic compartments. Salinity stress results in a massive K^+^ loss from the root cytosol mediated by a range of depolarization- and ROS-activated K^+^ channels [28,57]. To maintain normal metabolic activity, this cytosolic K^+^ depletion needs to be buffered, at the expense of the vacuolar pool, until plants activate additional K^+^ uptake systems, to regain lost K^+^ [29]. The vacuolar K^+^ pool reserves are estimated to maintain cytosolic K^+^ homeostasis for ~6 h [28] but need to be precisely regulated. Recently, Tang et al. [52] identified vacuolar TPK (two-pore K^+^) channels as a key player in this process in *Arabidopsis* and reported their regulation by CBL–CIPK interaction. Four CIPKs—CIPK3, 9, 23, and 26—were identified as partners of CBL2 and CBL3 that together regulate K^+^ homeostasis through activating vacuolar K^+^ efflux to the cytoplasm [52]. We believe that a similar scenario may be applicable to rice plants as well.

### 3.4. The Loss of Function of OsCIPK9 Results in a Hypersensitivity to ROS under Conditions of K^+^ Deficiency

Stress-induced calcium “signatures” are crucial for activation of plant adaptive cascades [3] and CBL–CIPK complexes operate as downstream Ca^2+^ sensors in this regulation [6,53]. As a result, activation of K^+^ uptake systems is tightly regulated by Ca^2+^ (e.g., AKT1 by CBL1–CIPK23 [56,58]). Previous studies on *Arabidopsis* showed that CIPK9 did not interact with any major plasma membrane-based K^+^ transporters such as AKT1, HAK5, AKT2, or SKOR [33]. However, whole-plant phenotypic observations are prone to possible misinterpretation, due to the functional redundancy of various CBL–CIPK members. Cell-based phenotyping offers better insights into mechanistic roles of plant kinases as regulators of membrane transport processes.

Both salinity and drought stresses result in accumulation of ROS species in plant tissues. Stress-induced ROS production is also reported in response to a broad range of other abiotic and biotic stresses. In this study, we showed that roots of *Oscipk9* also showed hypersensitivity to ROS under conditions of low K^+^ availability with a two-fold difference in the magnitude of H_2_O_2_-induced K^+^ efflux between mutant and WT plants (Figure 6). This difference disappeared when plants were exposed to adequate K^+^ supply. Consistent with these observations, also significant was the difference in the magnitude of ROS-induced Ca^2+^ fluxes between WT and *Oscipk9* roots (Figure 7) under low-K conditions. These results imply that the loss of function of OsCIPK9 results in a hypersensitivity to ROS and implements H_2_O_2_ signalling as a component of plant adaptive responses to low-K environment.

ROS and Ca^2+^ signals interact in a positive feedback manner forming self-amplifying loops composed of NADPH oxidase (encoded by RBOH genes) and ROS-activated Ca^2+^ channels [42]. Plants grown under conditions of K^+^ starvation possess higher basal levels of H_2_O_2_ [59], so activation of a “Ca^2+^-NADPH hub” will be more pronounced and rapid in this case. This is reflected in a bigger magnitude of ROS-induced Ca^2+^ uptake and K^+^ loss in low-K grown plants. The higher sensitivity of *Oscipk9* plants to H_2_O_2_ stimulation may be related either to the disturbance in Ca^2+^ sensing process or a possible role of CIPK9 in regulation of NADPH oxidase activity. The specific details of this process are the subject of separate studies. It should be also kept in mind that H_2_O_2_ is not the only ROS species produced in response to abiotic stresses. Other ROS types, such as superoxide or hydroxyl radicals, are also produced in various intracellular compartments, and might affect plant metabolism. In this context, understanding the role of CIPK9 in regulation of plant redox homeostasis and signalling warrants a separate investigation.

## 4. Materials and Methods

### 4.1. Plant Material and Growth Conditions

Seeds of rice plants, Oryza sativa L. Japonica cv Dongjin wild type, and its mutant Oscipk9, were obtained from Dr Chang-deok Han (National Institute of Agricultural Biotechnology, Seoul, Korea) and described in detail elsewhere [40]. Seeds were surface-sterilised with 1% *v*/*v* sodium hypochlorite (commercial bleach) for 10 min, and then thoroughly rinsed with sterile deionised water at least five times. Seeds were sown in sand, and then incubated at 28 °C and 100% relative humidity, and kept in darkness for five days, until germination. The seedlings were then transferred to a 5-litre hydroponic system, consisting of a number of light-tight black plastic containers, each holding 9 plants. Hoagland solution was used as the growth medium (1.25 mM KNO_3_; 0.5 mM Ca(NO_3_)_2_, 0.5 mM MgSO_4_; 42.5 μM Fe-EDTA; 0.625 mM KH_2_PO_4_; 0.16 μM CuSO_4_; 0.38 μM ZnSO_4_; 1.8 μM MnSO_4_; 45 μM H_3_BO_3_; 0.015 μM (NH_4_)_2_MO_7_O_24_; and 0.01 μM CoCl_2_ (pH 5.5–6.0)). Containers were placed into a climate-controlled glasshouse that was set on a light/dark cycle of 16/8 h and a day/night temperature of 28/20 °C, and relative humidity of ~80%. Two mercury vapour lamps (2 × 400 W) were set to provide 16-h days. The experiment design was a randomised block design with three replicates, with each container (replicate) holding nine plants for each treatment. The nutrient solution was changed every seven days. The rice seedlings were exposed to different abiotic stress conditions for 3 weeks, as described below. Experiments were conducted twice, with consistent results.

### 4.2. Treatments

Three types of experiments were conducted to study the effect of loss of function of *Oscipk9* on plant’s growth and development, under various environmental conditions. Firstly, eleven-day-old seedlings were exposed to two levels of salinity (moderate stress, 40 mM; and severe stress, 80 mM NaCl) for three weeks. In the second experiment, osmotic stress was implemented by addition of 11.8% (*w*/*v*) of polyethylene glycol 4000 (PEG4000) (isotonic to 80 mM NaCl), imposing an osmotic stress of 0.362 MPa. All treatments lasted for 3 weeks.

### 4.3. Whole-Plant Physiological Assessment

Chlorophyll content and stomatal conductance were measured on six randomly selected youngest fully expanded leaves of each treatment. All measurements were taken on a sunny day between 11:00 am and 1:00 pm, to minimize the diurnal influences. The chlorophyll content was measured using a Minolta Chlorophyll Meter SPAD-502 (Konica Minolta, Osaka, Japan); a Decagon leaf porometer (Decagon Devices Inc., Pullman, WA, USA) was used for the stomatal conductance measurements. Plants were then harvested, and their fresh weight was measured. Plants were then dried at 65 °C in a drier (Unitherm, Birmingham, UK) and their dry weight was recorded.

### 4.4. Osmolality and Ion Content

Leaf and root osmolality, and K^+^ and Na^+^ contents were determined using the freeze–thaw method. Harvested root samples were rinsed in 10 mM CaCl_2_ to remove the apoplastic Na^+^, then blotted dry on tissue paper. Next, the samples were placed into 1.5 mL microfuge tubes and stored at −20 °C for at least 24 h. The samples were subsequently thawed, and the sap squeezed from the tissues using a pointed glass rod. A small portion (10 µL) of these sap samples was used for osmolality determination using a vapour pressure osmometer (Vapo, Wescor Inc., Logan, UT, USA). The remainder of the sap samples were diluted ×100 times with distilled water and K^+^ and Na^+^ contents of the leaves and roots were measured using a flame photometer (Model PFP7 flame photometer, Jenway, Bibby Scientific Ltd., Staffordshire ST15 0SA, UK).

### 4.5. Non-Invasive Ion Flux Measurements

Net K^+^ and Ca^2+^ fluxes from roots were measured using the non-invasive MIFE microelectrode system (University of Tasmania, Hobart, Australia). All details on microelectrode fabrication and calibration, as well as the theory of MIFE ion flux measurements, are available from our previous publications [60,61]. Rice seeds were germinated inside an incubator set at 28 °C and 100% relative humidity. Four different combinations of potassium and calcium ions were used in the growth solution: low K^+^ (0.5 mM)/high Ca^2+^ (1.5 mM), low K^+^/low Ca^2+^ (0.1 mM), high K^+^ (50 mM)/high Ca^2+^, and high K^+^/low Ca^2+^. Roots of uniform and healthy 5–6-day-old seedlings were chosen and carefully placed on the centre of a glass holder and fixed firmly with Parafilm strips on both sides to avoid root movement during the measurement. The glass holder was then placed inside the measuring chamber that was partially filled with the bathing medium, BSM (Basal Salts Medium), consisting of 200 µM NaCl, 100 µM CaCl_2_, and 200 µM KCl. The pH level of the BSM solution was maintained at ~5.6. For conditioning, the roots were left in the bathing solution for approximately 30–60 min. The measuring chamber was then positioned on a microscope stage, and electrode tips aligned and positioned next to the root surface, at a distance of 50 µm. Basal net Ca^2+^ and K^+^ fluxes were recorded for 5–7 min from the mature root epidermis (ca 10 mm from the root tip). Then, 5 mM of H_2_O_2_ was administered to plants, and transient responses were recorded for another 25–30 min. The ion fluxes were then calculated using MIFEFLUX software, and the resulting data imported to an Excel spreadsheet for further analysis. To ensure consistency of results, plants were grown in several batches so data for each treatment came from plants grown in 3 or 4 batches (e.g., independent treatments).

### 4.6. Statistical Analysis

All data used in this paper are expressed as mean values ± SE. The statistical significance of mean and standard error values was determined by the *t*-test at *p* ≤ 0.05 using SPSS software version 20 (IBM support portal, Pullman, WA, USA).

## 5. Conclusions

The findings of this study revealed that the calcineurin B-like protein-interacting protein kinase 9 (CIPK9) is involved in regulation of K^+^ homeostasis in rice plants, particularly under the K^+^-deficient condition. The loss of function of CIPK results in a hypersensitivity to ROS in plant roots and mild salt- and osmotic-stress-tolerant phenotypes, attributed to the better control of stomata. Understanding the downstream signalling pathway and the role of CIPK9 in the cross-talks between ROS and ABA signalling may be instrumental for engineering plants with improved salinity and drought tolerance, to reduce impact of climate-driven abiotic stresses of crop production and food security.
